# Prevalence and Burden of Gait Disorders in Elderly Men and Women Aged 60–97 Years: A Population-Based Study

**DOI:** 10.1371/journal.pone.0069627

**Published:** 2013-07-24

**Authors:** Philipp Mahlknecht, Stefan Kiechl, Bastiaan R. Bloem, Johann Willeit, Christoph Scherfler, Arno Gasperi, Gregorio Rungger, Werner Poewe, Klaus Seppi

**Affiliations:** 1 Department of Neurology, Innsbruck Medical University, Innsbruck, Austria; 2 Department of Neurology, Radboud University Nijmegen Medical Center, Nijmegen, The Netherlands; 3 Department of Neurology, Hospital of Bruneck, Bruneck, Italy; Centre Hospitalier Universitaire Vaudois (CHUV), Switzerland

## Abstract

**Background:**

Although gait disorders are common in the elderly, the prevalence and overall burden of these disorders in the general community is not well defined.

**Methods:**

In a cross-sectional investigation of the population-based Bruneck Study cohort, 488 community-residing elderly aged 60–97 years underwent a thorough neurological assessment including a standardized gait evaluation. Gait disorders were classified according to an accepted scheme and their associations to falls, neuropsychological measures, and quality of life were explored.

**Results:**

Overall, 32.2% (95% confidence interval [CI] 28.2%–36.4%) of participants presented with impaired gait. Prevalence increased with age (p<0.001), but 38.3% (95%CI 30.1%–47.3%) of the subjects aged 80 years or older still had a normally preserved gait. A total of 24.0% (95%CI 20.4%–28.0%) manifested neurological gait disorders, 17.4% (14.3%–21.0%) non-neurological gait problems, and 9.2% (6.9%–12.1%) a combination of both. While there was no association of neurological gait disorders with gender, non-neurological gait disorders were more frequent in women (p = 0.012). Within the group of neurological gait disorders 69.2% (95%CI 60.3%–76.9%) had a single distinct entity and 30.8% (23.1%–39.7%) had multiple neurological causes for gait impairment. Gait disorders had a significant negative impact on quantitative gait measures, but only neurological gait disorders were associated with recurrent falls (odds ratio 3.3; 95%CI 1.4–7.5; p = 0.005 for single and 7.1; 2.7–18.7; p<0.001 for multiple neurological gait disorders). Finally, we detected a significant association of gait disorders, in particular neurological gait disorders, with depressed mood, cognitive dysfunction, and compromised quality of life.

**Conclusions:**

Gait disorders are common in the general elderly population and are associated with reduced mobility. Neurological gait disorders in particular are associated with recurrent falls, lower cognitive function, depressed mood, and diminished quality of life.

## Introduction

Gait disturbances and subsequent falls are among the major causes of chronic disability in the elderly population and, although being a common condition, so far have been subject to relatively few studies assessing overall frequency and clinical characteristics in community-based samples [1], [2]. The lack of data may partially derive from the absence of standard definitions and criteria for this heterogeneous group of disorders. Suggestions to classify gait abnormalities include syndromic approaches based upon the phenomenological features of gait and associated signs and symptoms [2]–[5] as well as etiological approaches asking for the neurological cause underlying the respective gait abnormality [3], [5]–[7]. Previous studies mainly used questionnaire-based assessments in the community or did examine gait objectively, but in a selected hospital-based outpatient cohort with gait disorders [1], [6]. Using a syndromic classification only, three studies involving samples from the Einstein Aging Study cohort found 16% to 20% of participants aged over 70-years suffering from neurological gait disorders [2], [4], [8]. However, these were community-based samples and not recruited as representative population samples [8]. Therefore, in the general elderly community, the prevalence of gait disorders still remains to be elucidated. It would also be helpful to establish the overall gait-related burden due to impaired mobility, falls, and overall decreased function. To achieve this, we performed specific investigations in the cohort of the ongoing population-based Bruneck Study [9]–[11].Our aims were to assess 1. the prevalence of neurological and non-neurological gait disorders according to a well-defined classification system accounting for sex differences and age trends, 2. the clinical types of (neurological) gait disorders and their respective severity assessed by quantitative gait measures, 3. the frequency of falls and their association to different types of gait disorders, 4. the association of gait disorders and falls to anxiety, depression, and cognitive decline, and 5. the impact of gait disorders on quality of life.

## Methods

### Participants

Our participants were recruited from the cohort of the Bruneck Study, a prospective population-based study on the epidemiology of cardiovascular and neurological diseases [9]–[11]. Bruneck is a small town situated in the alpine region of Northern Italy. Although inhabitants in this area are exclusively Caucasian, their Austro-German and/or Italian background contributes to heterogeneous geographic origins of the study participants. The study population was recruited as an age- and sex-stratified random sample of all inhabitants of Bruneck aged 40–79 years. Baseline assessment took place in 1990 and included 919 of 1000 persons selected from the official population register, spitted into age groups according to decades (125 women and 125 men in every decade from 40 to 79 years). Follow-up assessments were scheduled every five years. From the surviving 576 subjects, 488 returned for the last follow-up assessment in 2010 (follow-up rate 84.7%) and were used for the present cross-sectional study. Out of a total of 14 institutionalized subjects from the Bruneck Study cohort at that point in time, 11 were seen during the Bruneck Study 2010 and included in the present analysis. For the purpose of comparing age related differences, we divided our population in three subgroups of 60–69 (n = 205), 70–79 (n = 163), and 80–97 (n = 120) years of age.

### Ethics

The study protocol was approved by the local ethics committee (institutional review board of the local medical service of south Tyrol, Bolzano, Italy) and all participants gave written informed consent. All investigations were carried out in accordance to the principles expressed in the Declaration of Helsinki.

### Procedures

The study protocol included a clinical neurological assessment with emphasis on movement disorders. The interviews and examinations were carried out by two neurologists (one senior neurologist with special expertise in movement disorders and one fellow in the field of movement disorders). Firstly, a careful general history, including family- and past medical history, was taken and screening for secondary causes of disease, especially movement disorders including drug-exposure was performed. Standard interviews sought for clinical symptoms suggestive of gait disorders such as slowness of movements, difficulties of gait (insecurity, scuffing shoes, short steps, and getting glued feet to the floor) feeling of unsteadiness and imbalance, fear of falling, and the frequency of falls over the past year. Falls, also ascertained from history, were defined as a subject unintentionally coming down on the floor not due to a major intrinsic or extrinsic event [12]. Recurrent falls were defined as more than one fall within the last year. Cognitive performance was measured by the Mini Mental State Examination (MMSE). Mood was evaluated by the Beck Depression Inventory (BDI) and the Hospital Anxiety and Depression Scale (HADS-Anxiety and HADS-Depression). Quality of life (QoL) was assessed using the multidimensional World Health Organization QoL BREF (WHOQoL-BREF) instrument.

Subsequently, all participants underwent a standardized neurological examination including the UPDRS-III (Unified Parkinson’s Disease Rating Scale, motor section). Classification of participants with clinical signs of parkinsonism was done according to current diagnostic criteria for parkinsonian disorders [13]. Due to a lack of standard definition or criteria for the rather heterogeneous group of gait disorders, we decided to resort to a well-defined classification system recently published by Snijders and colleagues [3]. It consists of a practical approach based on a three step model. Step one leads to a possible, clinically based gait disorder. Within this first step, three elements were captured: (1) core gait features were assessed while observing participants walking for 10 meters, turning, and walking back seeking for e.g. high steppage with dropping foot, (bilateral) circumduction, staggering and wide-based gait, shuffling gait, hesitation and freezing, ‘walking on ice’, etc.; (2) additional clinically based gait and balance tests regularly included the Unterberger-, the Romberg’s-, and the pull test, the tandem-walk and walking while keeping eyes closed to test for balance and postural stability, the heel- and toe walk to test for strength and the stop walking while talking tool to test for aggravation upon performance of a secondary task; (3) associated symptoms and signs were assessed during the general neurological evaluation seeking for e.g. lower motor neuron features, disturbed proprioception, cerebellar ataxia, parkinsonism, frontal release signs, etc. Step two includes ancillary investigations, response to treatment, and prolonged follow-up and leads to a probable diagnosis. As this study was performed in the Bruneck hospital, information about potential reasons for gait impairment was available (e.g. brain imaging, glucose, and HbA1c levels, vitamin B12 deficiency, response to dopaminergic treatment, longitudinal clinical data, etc.) and a probable etiological diagnosis of gait impairment could be made. The classification included nine neurological gait disorders: I paretic/hypotonic, II spastic, III sensory ataxic, IV cerebellar ataxic, V vestibular ataxic, VI dyskinetic, VII hypokinetic-rigid (parkinsonian), VIII cautious, and IX higher-level [3]. Gait disorders resulting from more than one neurological condition were classified and reported as ‘multiple’ and the contributing elements were noted. Step three consists of a post-mortem examination leading to a definite diagnosis and was not performed. Apart from these neurological causes, we subsumed orthopaedic, including an antalgic gait (due to e.g. joint pain), and ophthalmologic reasons for gait impairment as non-neurological causes. Gait disorders resulting from both neurological and non-neurological causes were classified and reported as ‘combined’. Impairment was defined as mild (no assistance required), moderate (dependent on walking aid) or severe (non-ambulant) [2]. As quantitative gait measures, the Hauser ambulation index [14], the Tinetti balance and gait scale [15], and the Timed Up and Go test [16] were applied. Overall gait speed was measured during undisturbed straight walking at the subject’s own comfortable speed over a distance of 8 meters using a simple stop watch.

### Inter-rater Reliability

To assess the reliability of gait evaluations, an independent sample of 43 inpatients at the department of neurology from the Innsbruck Medical University with distinct neurological diagnoses were examined by the two study clinicians blinded to the results of each other. There was high agreement between the two assessments in the classification of the various gait disorders (kappa ranging from 0.78 to 0.99, each p<.001). Overall unweighted kappa was 0.95 (p<0.001). Therefore a good inter-rater reliability was achieved.

### Statistical Analysis

Data were tabulated and analyzed using SPSS 20.0 for windows (SPSS Inc., Chicago, IL). Prevalence rates are given in percent of the respective category. Ninety-five percent confidence intervals (95%CI) were calculated using the modified Wald method [17]. The chi-square test was used for gender and age category distribution. As data from the qquantitative gait measures (Hauser index, Tinetti scale, Timed Up and Go test and gait speed) were not normally distributed, group comparisons for these severity measures were performed with Mann-Whitney-U tests and post-hoc Bonferroni corrections. Impact of various gait disorders and the quantitative gait measures on the occurrence of recurrent falls as well as associations of age, gender, cognition, and mood on the occurrence of gait disorders, neurological gait disorders, and recurrent falls were assessed with an unconditional logistic regression analysis. Associations were expressed by the odds ratio (OR). For continuous variables (age, Hauser index, Tinetti scale, Timed Up and Go test, gait speed, MMSE, BDI, HADS-Anxiety, and HADS-Depression) ORs were calculated for a one standard deviation unit change in variable levels in order to render the odds comparable. The significance level was set at p<0.05.

## Results

### Prevalence of Gait Disorders

Of the 488 participants, 142 reported difficulties while walking (29.1%; 95%CI 25.2%–33.3%), but 18 of them (3.7%; 2.3%–5.8%) showed no gait abnormalities during the neurological examination at the time of the visit. Overall, 157 participants (32.2%; 95%CI 28.2%–36.4%) had a gait disorder upon examination, 33 of these subjects were not aware of it (6.8%; 4.8%–9.4%). The frequency of gait disorders was strongly age-related (p<0.001; Table 1). Gait disorders tended to be more prevalent in women than in men (p = 0.053) and women more frequently reported difficulties while walking (p<0.001).

**Table 1:** Prevalence of gait disorders, according to sex and age.

Sex	Age category	Total No.	GD No., % (95%CI)		Neurological No.	Combined No.	Non-neurological No.
Men^a^	60–69	103	12	11.7 (6.7–19.4)	6	3	3
	70–79	79	27	34.2 (24.7–45.2)	14	4	9
	80–97	45	24	53.3 (39.1–67.1)	14	7	3
	Total	227	63	27.8 (22.3–33.9)	34	14	15
Women^a^	60–69	102	10	9.8 (5.2–17.3)	2	2	6
	70–79	84	34	40.5 (30.6–51.2)	10	10	14
	80–97	75	50	66.7 (55.4–76.3)	26	19	5
	Total	261	94	36.0 (30.4–42.0)	38	31	25
Men and Women	60–69^b^	205	22	10.7 (7.1–15.8)	8	5	9
	70–79^b^	163	61	37.4 (30.4–45.1)	24	14	23
	80–97^b^	120	74	61.7 (52.7–69.9)	40	26	8
	Total	488	157	32.2 (28.2–36.4)	72	45	40

Abbreviations: GD, gait disorders.

a Significance for sex difference: p = 0.053;

b significance for age trends: p<0.001.

### Types and Severity of Gait Disorders

As outlined in Table 1, 117 subjects showed a neurological gait disorder and 85 a non-neurological gait disorder. Of these, 45 subjects had combined reasons for their gait impairment. A detailed breakdown of neurological gait disorders is represented in Table 2; 81 subjects suffered from a distinct entity and 36 from multiple neurological gait disorders. In the latter group single combinations were close to the proportions expected by chance, except for the combination of higher-level and sensory ataxic gait. There was no association of neurological gait disorders with gender. Within this group, however, the cautious walking pattern was associated to female gender (p = 0.017). Also, non-neurological gait disorders were associated with female gender (p = 0.012). Non-neurological gait disorders occurred due to osteoarthritis of the knee (n = 28), and the hip (n = 23) and various other orthopaedic reasons in the remaining subjects as well as ophthalmological reasons leading to visual disturbances in 12 cases. All 11 institutionalized subjects had a neurological gait disorder, 7 in combination with a non-neurological gait disorder.

**Table 2:** Prevalence of the various neurological gait disorders.

Neurological GD	No.(#)	% of neurologicalGD (95%CI)	TotalNo. (§)	Causes (No.)
Single neurological	81	69.2 (60.3–76.9)		
Sensory ataxic	22	18.0 (12.7–26.9)	46	Peripheral sensory neuropathy (46)^b^
Parkinsonian	19	16.2 (10.6–24.1)	34	18 definite Parkinson’s disease (18), drug-induced parkinsonism (8),dementia with parkinsonism (4), parkinsonism (4)^c^
Higher level	9	7.7 (3.9–14.2)	31	CT-scan confirmed vascular encephalopathy (20) and normalpressure hydrocephalus (1), severe dementia (7), hypoxic ischemicencephalopathy (1), clinical gait apraxia of unknown reason (2)
Cerebellar ataxic	7	6.0 (2.7–12.0)	10	Cerbellar stroke (3), cerebellar lesion due to multiple sclerosis (1),severe essential termor (3), post-vaccinal cerebellitis (1), chronicalcohol abuse (1), multiple system atrophy (1)
Cautious	7	6.0 (2.7–12.0)	7	Idiopathic, associated to fear of falling (7)^d^
Paretic/hypotonic	6	5.1 (2.1–11.0)	14	Neurogenic claudication (7), diabetes type 2 neuropathy (1), nerveleasion due to trauma (3) or surgery (1), distal paraparesis as residualafter Guillain–Barré syndrome (1), unknown (2)
Spastic	6	5.1 (2.1–11.0)	7	Ischemic stroke (3), intracerebral hemorrhage (3), congenital (1)
Vestibular ataxic	4	3.4 (1.1–8.8)	6	Bilateral vestibulopathy (3), recent vestibular neuronitis (1),recent Meniere's attack (1); acoustic neuroma with surgery (1)
Dyskinetic	1	0.9 (0.0–5.2)	4	levodopa-induced dyskinesia (3), chorea (1)
Multiple Neurological GD^a^	36	30.8 (23.1–39.7)		
Total	117			

The table lists No (#) and Total No (§). No (#) stands for gait disorders, which were single entities in 81 subjects and multiple gait disorders due to combinations of different entities in 36 subjects. Total No (§) stands for the total number of subjects presenting with a specific gait disorder. For the subjects with multiple gait disorders their contributors are listed additionally in the legend (see ^a^). E.g. there are 22 subjects who presented with sensory ataxia as a single neurological gait disorder [i.e. No (#)] and another 24 subject with a sensory ataxic gait in combination with other neurological gait disorders adding up to a total of 46 subjects with this gait abnormality [i.e. Total No (§)].Abbreviations: GD, gait disorders.

a Contributors to multiple neurological GD (No.): Sensory ataxic (24), higher level (22), parkinsonian (15), paretic/hypotonic (8), cerebellar ataxic (3), vestibular ataxic (2), dyskinetic (3), spastic (1);

b Diagnosed in the presence of neuropathic symptoms (numbness, altered sensation, or pain in the feet) and neurologic signs (decreased ankle reflexes, decreased distal sensation, and disturbed vibration sense) according to current criteria [30];

c Subjects with a parkinsonian syndrome not fulfilling criteria for definite Parkinson’s disease [13];

d With core gait features of a slow, wide base gait with short steps like ‘walking on ice’ [3].

Quantitative gait measures for the different gait disorders are shown in Table 3. All used measures significantly display gait impairment in all neurological gait disorders compared to healthy subjects (p<0.001), except for the Timed Up and Go test and gait speed in vestibular and cerebellar ataxic gait, where all subjects had a mild severity of the gait disorder. Greatest impairment was found in spastic and higher-level gait, and in multiple neurological gait disorders, where over one third were moderately to severely affected. Also pure non-neurological gait disorders lead to significant gait impairment, which was even higher when combined with a neurological gait disorder (p<0.001).

**Table 3:** Gait disorders and association to gait measures.

Gait disorders (No.)	Hauser index [0–8]	Tinetti score [0–28]	Timed up and go [sec]	Gait speed [cm/sec]	Severity (%)^a^
One neurological (81)	2.4 (1.7)	21.0 (5.8)	14.6 (6.5)	87.0 (25.6)	16
Sensory ataxic (22)	2.3 (1.8)	21.5 (6.0)	13.5 (6.0)	88.7 (24.6)	18
Parkinsonian (19)	2.1 (1.6)	22.1 (1.7)	15.6 (3.7)	88.6 (21.1)	16
Higher Level (9)	3.4 (2.5)	17.1 (8.7)	19.3 (8.1)	81.1 (31.0)	33
Cerebellar ataxic (7)	2.0 (0.6)	22.3 (2.8)	10.4 (2.2)	100.6 (35.9)	0
Cautious (7)	2.3 (1.1)	21.6 (4.0)	13.9 (3.2)	80.0 (22.0)	14
Paretic/hypotonic (6)	2.3 (1.4)	20.1 (4.8)	16.4 (9.3)	86.9 (31.8)	17
Spastic (6)	2.8 (1.8)	17.3 (5.8)	23.7 (12.8)	69.8 (28.5)	33
Vestibular ataxic (4)	1.8 (1.0)	22.8 (1.7)	10.6 (0.6)	91.3 (18.4)	0
Dyskinetic (1)					0
Multiple Neurological (36)	3.7 (2.4)	15.9 (7.6)	17.2 (7.4)	71.8 (30.9)	44
Total neurological (117)	2.8 (2.0)	19.4 (6.8)	15.5 (6.9)	82.7 (28.0)	25
Only neurological GD (72)	2.4 (1.8)	21.0 (5.8)	13.8 (4.9)	86.0 (26.6)	15
Only non-neurological GD (40)	1.9 (1.2)	23.7 (3.9)	12.8 (4.5)	93.4 (23.9)	15
Combined (45)	3.4 (2.2)	16.9 (7.6)	18.5 (8.7)	77.1 (29.6)	40
Total GD	2.6 (1.9)	20.5 (6.4)	14.8 (6.4)	85.4 (27.3)	22
No GD (331)	0.1 (0.3)	28.0 (0.2)	8.9 (1.6)	122.3 (22.2)	–

Abbreviations: GD, gait disorders.

Results of the Hauser index, Tinetti score, Timed up and go test and gait speed are reported in means (SD, standard deviation).

a Proportion of subjects with a moderate (dependent on walking aid) or severe (non-ambulant) gait disorder.

### Falls

Overall, 45 subjects (9.2%; 95%CI 6.9%–12.1%) suffered from recurrent falls over the past year (32 women and 13 men; p = 0.018) and all of them had a gait disorder. Thus, of the subjects with gait disorders 28.7% (95%CI 22.2%–36.2%) fell recurrently and 20.4% (14.8%–27.4%) reported fear of falling (29 women and 3 men; p<0.001), including the 7 subjects with a cautious gait. Mean frequency of falls was 0.8 per month, ranging from twice a year to once a day. Table 4 presents the associations of various types of gait disorders with recurrent falls; neurological gait disorders, but not non-neurological gait disorders were significantly associated to recurrent falls. Also quantitative gait measures were significantly associated with falls, particularly reduced gait speed.

**Table 4:** Odds ratios of recurrent falls for the various types of gait disorders and gait speed.

Variable	FallersNo.	TotalNo.	OR	(95%CI)	P-value
Sensory ataxic	18	46	3.7	(1.5–9.3)	0.006
Parkinsonian	15	34	5.3	(2.0–14.2)	0.001
Higher level	16	31	3.2	(1.1–9.4)	0.030
Cerebellar ataxic	4	10	3.0	(0.4–21.0)	0.262
Cautious^a^	1	7	–		–
Paretic/hypotonic	5	14	2.9	(0.7–11.6)	0.124
Spastic	4	7	20.9	(2.2–202.4)	0.009
Vestibular^a^	1	6	–		–
Dyskinetic^a^	1	4	–		–
One neurological GD	21	81	2.4	(1.0–5.4)	0.039
Multiple neurological GD	20	36	7.1	(2.7–18.7)	<0.001
Only neurological GD	22	72	3.3	(1.4–7.5)	0.005
Only non neurological GD	4	40	1.4	(0.4–5.1)	0.655
Combined GD	19	45	4.1	(1.6–10.2)	0.003
Hauser index^b^			4.7	(2.8–7.7)	<0.001
Tinett score^b^			3.9	(2.5–6.1)	<0.001
Timed Up and Go test^b^			2.7	(1.6–4.3)	<0.001
Gait speed^b^			5.5	(2.8–11.0)	<0.001

Abbreviations: GD, Gait disorders; OR, odds ratios.

OR are calculated by logistic regression analysis and corrected for age, gender and MMSE-scores.

a Numbers too low in the respective category for the calculation of the ORs.

b For continuous variables ORs were calculated for a one standard deviation unit change in variable levels in order to render odds comparable.

### Neuropsychological Associations of Gait Disorders and Falls

A logistic regression analysis revealed that age, lower mental function and depression (but not anxiety) were significantly associated with gait disorders, in particular neurological gait disorders and recurrent falls (Table 5). Correction for age and gender did not alter these results.

**Table 5:** Association of age, gender, cognition and mood with gait disorders, neurological gait disorders and recurrent falls.

Variable	Unadjusted		Adjusted for age and gender	
	OR (95%CI)	P-value	OR (95%CI)	P-value
**Gait disorders (n = 157)**				
Age	3.4 (2.7–4.4)	<0.001	–	–
Gender (w:m)	1.5 (1.0–2.2)	0.052	–	–
MMSE (decline)	3.0 (2.2–4.1)	<0.001	1.8 (1.3–2.5)	<0.001
BDI	1.7 (1.3–2.3)	<0.001	1.6 (1.2–2.2)	0.002
HADS-Anxiety	1.1 (0.9–1.4)	0.266	1.1 (0.9–1.4)	0.477
HADS-Depression	1.7 (1.4–2.1)	<0.001	1.5 (1.2–1.9)	0.001
**Neurological gait disorders (n = 117)**				
Age	3.9 (2.9–5.1)	<0.001	–	–
Gender (w:m)	1.3 (0.9–2.0)	0.173	–	–
MMSE (decline)	3.6 (2.6–4.9)	<0.001	2.0 (1.5–2.9)	<0.001
BDI	1.8 (1.4–2.4)	<0.001	1.8 (1.3–2.4)	0.001
HADS-Anxiety	1.1 (0.9–1.4)	0.229	1.1 (0.9–1.4)	0.349
HADS-Depression	1.8 (1.4–2.2)	<0.001	1.5 (1.2–2.0)	0.001
**Recurrent falls (n = 45)**				
Age	4.5 (3.0–6.9)	<0.001	–	–
Gender (w:m)	2.3 (1.2–4.4)	0.018	–	–
MMSE (decline)	2.2 (1.6–3.0)	<0.001	1.5 (1.1–2.0)	0.019
BDI	1.9 (1.3–2.7)	0.001	1.8 (2.0–2.7)	0.005
HADS-Anxiety	1.1 (0.8–1.6)	0.410	1.0 (0.7–1.5)	0.901
HADS-Depression	1.9 (1.5–2.5)	<0.001	1.5 (1.1–2.1)	0.011

Abbreviations: MMSE, Mini Mental State Examination; BDI, Beck Depression Inventory; HADS, Hospital Anxiety and Depression Scale; OR, odds ratio.

ORs are calculated by logistic regression analysis. For continuous variables (age, MMSE, BDI, and HADS) ORs were calculated for a one standard deviation unit change in variable levels in order to render odds comparable.

### Quality of Life

WHOQoL-BREF scores are diminished in subjects with gait disorders, in particular neurological gait disorders, for general QoL and quality of health as well as in the domains physical health, psychological health and environment, but not in the domain social relationships (Table 6).

**Table 6:** Association of gait disorders and neurological gait disorders with quality of life.

	General	Physical health	Psychological health	Social relationships	Environment
All GD	68.4 (±15.0)p<0.001	76.4 (±11.9)p<0.001	72.4 (±14.0)p = 0.005	71.6 (±12.6)ns	82.3 (±9.9)p = 0.038
Neurological GD	67.0 (±15.0)p<0.001	75.2 (±12.3)p<0.001	70.6 (±14.3)p = 0.001	70.3 (±11.4)ns	81.3 (±9.9)p = 0.009
No GD	76.6 (±12.3)	84.7 (±8.7)	77.9 (±11.5)	73.1 (±12.2)	84.9 (±9.5)

Abbreviations: GD, Gait disorders.

The self administered WHO Quality of Life-BREF questionnaire assesses the general quality of life and health as well as the QoL in the four domains physical health, psychological health, social relationships, and environment. Results are reported in mean transformed scores (where 100 points represent maximum of respective item, ± standard deviation); P values refer to differences to the group without GD and are corrected for age and gender.

## Discussion

In this unselected population-based cohort representing the general elderly community, we detected a high overall prevalence of gait disorders, affecting one third of our subjects with a marked age-dependent increase. Gait was carefully and comprehensively evaluated according to an established [3] and further standardized protocol, with a combination of detailed history taking, detailed gait assessment including core gait features plus a standard set of gait and balance tests, general neurological examination, and the results of ancillary studies. Consequently, gait disorders were classified syndromically and etiologically according to an accepted scheme [3], separating clearly defined neurological gait disorders from non-neurological gaits, or combinations thereof. In the Bruneck Study cohort, 24.0% of participants had a neurological gait problem, 17.4% suffered from non-neurological gait disorders, and 9.2% had combinations of both. Within the subgroup of neurological gait disorders, 69.2% suffered from distinct neurological entities and 30.8% had multiple neurological causes. The sensory ataxic, higher-level and parkinsonian gait were the most common subtypes. The high prevalence of sensory ataxic gait represents an understandable finding, considering the fact that peripheral sensory neuropathy is common in the elderly and may easily affect gait function [6], [18]. White matter lesions can underlie higher-level gait disorders and these white matter lesions are associated with vascular risk factors such as advanced age, diabetes, and obesity [3], [19], [20]. The same factors can also contribute to a sensory gait ataxia [18], explaining the frequent coexistence of these two conditions in our sample. The high frequency of parkinsonian gait features in the elderly has already been described [6], [21]. However, our detailed diagnostic approach additionally revealed that such gait emerged mostly due to PD, but also due to secondary parkinsonism. Severity measures displayed the greatest functional impairment in spastic and higher level gait, as well as in multiple neurological gait disorders. Subjects with cerebellar ataxic and vestibular gait were less impaired. These participants were younger and all of them were mildly affected. The overall trend of a higher prevalence of gait disorders in women could be explained by a significant higher prevalence of non-neurological gait disorders in women. A plausible contributor to this female preponderance may be the higher prevalence of joint-pain in women as reported in literature [2], [22]. Among the neurological gait disorders only the cautious walking pattern was significantly associated to female gender. This is in line with our and previous findings in the community showing a higher prevalence of fear of falling in women [23].

Recurrent falls occurred in one tenth of our cohort, whereas community-based studies found that one sixth of the population aged 65 years or older experience recurrent falls [24]. The relatively lower fall rate may be explained by the lower age of our subjects, but might, however, also represent an underestimation as we relied upon a single interview to determine fall-frequency. Nevertheless, fall-related results seem to be accurate as female gender and slow gait were significantly associated to the risk of falls as previously reported [8], [25]. Interestingly, all fallers had a gait disorders and subjects with a neurological gait disorders were three times more likely to suffer from recurrent falls. Among neurological gait disorders the parkinsonian, sensory ataxic, spastic, higher level gait, and particularly multiple neurological gait disorders were significantly associated with recurrent falls. The same occurred for reduced gait performance as measured with the objective gait tests, particularly reduced gait speed. Therefore, timely gait assessment is likely to identify subjects at increased risk of falling. Gait is a marker for otherwise undetected and perhaps treatable disease and falls could be prevented or postponed in these subjects using a tailored treatment approach. Furthermore, among the subjects aged over 80 years, 38.3% still had a normally preserved gait, underlining that gait impairment must not be accepted as an inevitable concomitant of aging.

Although we have not applied an extensive neuropsychological testing battery, we could detect a significant association of lower cognitive function and higher depression scores with gait disorders and falls. Deficits in executive functions are known to influence gait [26], [27], [28] and may represent prodrome to falls [29]. Therefore, the lower MMSE-scores in our subjects with gait disorder may display a cognitive impairment and indicate a higher risk for falls.

Another important novel finding of this study is that QoL scores, as assessed by the generic measure WHOQOL-BREF, are significantly impaired in subjects with gait disorders. The worst affected areas compared to subjects without gait disorders were those related to physical and psychological health and to a lesser extent environmental functioning, whereas social relationship was not impaired. Future studies should assess predictors and correlates of QoL in subjects with gait disorders.

Our assessment has several strengths including the relatively large cohort of the Bruneck Study, representing a typical elderly western population with respect to demographic and lifestyle characteristics. Gait was carefully and comprehensively evaluated according to an established [3] and further standardized protocol, allowing for an accurate syndromic and etiological categorisation of gait disturbances according to an accepted scheme [3]. Inter-rater reliability of this classification system was high. There are, however, limitations to this study. The population size of 488 leads to rather wide confidence intervals and does not allow a precise estimate of rare diseases’ prevalence. Although participation rate was high with nearly 85%, we cannot exclude significant differences between participating subjects and those who did not with regard to presence of gait disorders and falls. Nevertheless, due to the population-based nature of the Bruneck Study, the relative contributions of the various types of gait disorders are likely to be an accurate reflection of real life. Prevalence rates obtained here, however, do not necessarily apply to other ethnic groups and might vary in different populations. Also, our study lacks an evaluation of gait features with the help of videotaping or kinematic assistances. However, the merits of such techniques, in particular the objective kinematic measures, compared to gait-assessment by an experienced clinician remain to be established. Finally, the cross-sectional nature of the present study did not allow for a prospective assessment of the gait-related burden. Further follow-ups of this well described cohort will give additional information on the association of gait disorders with potential serious complications such falls and fractures as well as nursing home placement and mortality.

To our knowledge, there is no previous study reporting the prevalence, distribution and clinical characterisation of gait disorders and their functional correlates in a cohort representative for the general community. Our study suggests that gait disorders are common in the general elderly population and are associated with reduced mobility. Neurological gait disorders in particular are associated with recurrent falls, lower cognitive function, depressed mood, and diminished QoL. The results of our study should increase awareness among clinicians and health authorities to the high prevalence and associated disease burden of gait disorders, in particular neurological gait disorders, in the general elderly community.
